# Empathy Mediates the Effects of Age and Sex on Altruistic Moral Decision Making

**DOI:** 10.3389/fnbeh.2016.00067

**Published:** 2016-04-12

**Authors:** Jan B. Rosen, Matthias Brand, Elke Kalbe

**Affiliations:** ^1^Institute of Gerontology, Psychological Gerontology and Center for Neuropsychological Diagnostics and Intervention, University of VechtaVechta, Germany; ^2^General Psychology: Cognition and Center for Behavioral Addiction Research, Department of Computer Science and Applied Cognitive Science, University of Duisburg-EssenDuisburg, Germany; ^3^Erwin L. Hahn Institute for Magnetic Resonance ImagingEssen, Germany; ^4^Neuropsychology and Gender Studies and Center for Neuropsychological Diagnostics and Intervention, Department of Medical Psychology, University Hospital CologneCologne, Germany

**Keywords:** moral decision making, aging, sex, empathy, reasoning, theory of mind

## Abstract

Moral decision making involves affective and cognitive functions like emotional empathy, reasoning and cognitive empathy/theory of mind (ToM), which are discussed to be subject to age-related alterations. Additionally, sex differences in moral decision making have been reported. However, age-related changes in moral decision making from early to late adulthood and their relation to sex and neuropsychological functions have not been studied yet. One hundred ninety seven participants (122 female), aged 19–86 years, were tested with a moral decision making task comprising forced choice “everyday life” situations in which an altruistic option that favors a socially accepted alternative had to be considered against an egoistic option that favors personal benefit over social interests. The percentage of altruistic decisions was analyzed. A structural equation model (SEM) was calculated to test the hypothesis whether age and sex predict altruistic moral decision, and whether relevant neuropsychological domains mediate these hypothesized relationships. A significant relationship between age and moral decision making was found indicating more frequent altruistic decisions with increasing age. Furthermore, women decided more altruistically than men. The SEM showed that both age and sex are significant predictors of altruistic moral decision making, mediated by emotional empathy but not by reasoning. No cognitive empathy and ToM scores were correlated to age and moral decision making at the same time and thus were not included in the SEM. Our data suggest that increasing age and female sex have an effect on altruistic moral decisions, but that this effect is fully mediated by emotional empathy. The fact that changes of moral decision making with age are mediated by emotional empathy can be interpreted in the light of the so-called “positivity effect” and increasing avoidance of negative affect in aging. The mediated sex effect might represent both biological aspects and socialized sex roles for higher emotional empathy leading to more altruistic decisions.

## Introduction

Moral decision making is based on the complex integration of affective and cognitive processes (e.g., Greene et al., 2004; Decety et al., 2011; Patterson et al., 2012). Although aging is accompanied by changes of affective and cognitive processes (Glisky, 2007; Ardelt, 2011; Charles, 2011), research on moral decision making in healthy participants has mainly focused on childhood and young adulthood (e.g., Decety et al., 2011). The aim of the current study is to examine moral decision making from early to late adulthood and to define the influence of age-related changes in affective and cognitive processing. Based on recent evidence for sex differences in moral decision making (Fumagalli et al., 2010a,b; Youssef et al., 2012; Migliore et al., 2014), sex and its relation to age is also investigated.

Moral decision making comprises decisions that are subject to generally accepted and culturally shaped moral norms of behavior (Reynolds and Ceranic, 2007; Moll and Schulkin, 2009). Thus, disadvantageous moral decision making has socially unfavorable outcomes (e.g., Haviv and Leman, 2002) or even involve legal consequences (e.g., Blair, 1995).

Affective functions substantially contribute to moral decision making (Haidt, 2001); it is modulated by an individual’s affective state (Polman and Ruttan, 2011) and emotional awareness (Koven, 2011). Evidence on a physiological level is given by Starcke et al. (2011), who induced stress to a sample of healthy participants and found that the increase of cortisol is related to egoistic moral decisions in high emotional, but not in low emotional moral conflicts. Aversive emotional reactions related to stress seem to interfere with positive affect that was found associated with altruistic moral decisions (Starcke et al., 2011). Therefore, situational emotionality and intra-individual affective processing seem to be important for the modulation of moral decisions. In particular, emotional empathy, i.e., the spontaneous emotional reaction to experiences of other people (Davis, 1983), considerably contributes to moral decision making (e.g., Decety et al., 2011; Gleichgerrcht and Young, 2013). More specifically, Batson (2010) found that empathic concern, as a measure of emotional empathy, motivates altruistic behavior and hereby highlights prosocial goals. There is some evidence for differences between younger and older adults regarding empathic concern on the one hand and moral decision making on the other hand (e.g., Gleichgerrcht and Young, 2013). However, evidence for a correlational relationship between emotional empathy and moral decision making rather than group differences is scarce (Gleichgerrcht and Young, 2013). Furthermore, the existing data on the role of emotional empathic reactions in the context of moral decisions was assessed for extreme moral dilemma situations (i.e., situations that deal with matters of life and death) and its validation for everyday moral situations hitherto is an open issue.

With regard to cognitive processes, executive control has been related to difficult moral decisions in extreme dilemmas that require moral tradeoffs in situations with high personal involvement, e.g., actively pushing a stranger off a bridge in order to save five others (Greene et al., 2004). In these decisions, executive control was proposed to support the overcoming of salient and intuitive options (Kahane et al., 2012). Reasoning abilities are important to exercise executive control in moral situations, because reasoning is substantially involved in cognitive conflict resolution and emotion regulation (Patterson et al., 2012). In this respect, reasoning can profoundly shape moral permissibility ratings (Paxton et al., 2012) and help to reflect on emotional responses to external contingencies (Koven, 2011; Tassy et al., 2012), like anticipated personal and impersonal consequences (Jeurissen et al., 2014). Whereas reasoning and executive control seem to facilitate utilitarian decisions in extreme moral dilemmas (Greene et al., 2004; Paxton et al., 2012), one could suppose that in everyday moral situations egoistic options are promoted, because egoistic decisions provoke aversive affective states that need to be overcome (Sommer et al., 2010; Rosen et al., 2013). However, no relationship between reasoning and egoistic everyday moral decisions has been reported so far.

Additionally, theory of mind (ToM) – the ability to infer mental states of others (Adolphs, 2001) – has been shown to be relevant for moral decision making (Young et al., 2010; Zalla et al., 2011). Especially permissibility ratings of intentional and accidental moral transgressions are modulated by ToM (Killen et al., 2011) so that intentional harm is condemned stricter (Young et al., 2010). Also, the pursuit of one’s own personal interest can be facilitated by ToM (Press and Dyson, 2012; Weiland et al., 2012), and ToM might promote egoistic decisions in everyday moral conflict situations (Rosen et al., 2013). In contrast, increased activation of ToM-related brain areas was associated with altruistic everyday moral decisions in adolescents (Sommer et al., 2014). Thus, the question remains how ToM influences everyday moral decision making.

Age-related affective and cognitive changes from young to late adulthood relate to functions that are associated to moral decision making. There are differences between younger and older adults in their experience and regulation of affective states. Elderly people report stronger emotional arousal if stressors cannot be avoided (Stawski et al., 2008). On the other hand, they show more effective coping strategies to manage these stressors (e.g., Charles et al., 2009; Grossmann et al., 2010). Furthermore, motivational changes relate to affective processing of older adults. In detail, older people show a tendency to avoid negative and approach positive stimuli (Charles et al., 2009), also referred to as “positivity effect.” The positivity effect has been discussed in the context of evidence for a relative stability of emotional well-being in older adults despite physical and cognitive deterioration (termed “aging paradox,” Charles and Carstensen, 2010). Accordingly, older people more often try to de-escalade interpersonal conflict situations (Grossmann et al., 2010) and show greater acceptance of negative emotions than younger adults (Shallcross et al., 2013). Evidence for age-related changes of emotional empathy has been reported, with controversial results concerning the question whether emotional empathy increases (Gould and Macneil Gautreau, 2014), declines (May and Alligood, 2000), or remains stable with advancing age (Bailey et al., 2008), or both variables show an inverse-*U*-shaped relationship (O’Brien et al., 2013). Despite these controversial findings, it was recently shown that emotional empathic reactions more strongly motivates older than younger adults to exhibit prosocial behavior (Beadle et al., 2015). The relation between age, emotional empathy and everyday moral decision making has not been investigated in one comprehensive experimental design yet.

Cognitive aging is characterized by a decline of episodic memory, working memory and executive functions (e.g., Glisky, 2007; Phillips et al., 2011). Age-related non-moral decision making difficulties seem to be related to decline in global cognitive functioning and executive functions (Brand and Markowitsch, 2010). Executive functions are composed of a heterogeneous set of subfunctions that substantially serve the exercise and maintenance of cognitive control (Patterson et al., 2012; Diamond, 2013). In this context, age effects on cognitive flexibility and reasoning (e.g., Borghesani et al., 2013) are of special interest (Paxton et al., 2012), because the resolution of cognitive conflict and regulation of emotional arousal induced by moral situations (Greene et al., 2004; Patterson et al., 2012) are likely to be affected by age-related alterations of executive functions and reasoning (Paxton et al., 2012), e.g., concerning the overcoming of moral intuitions (Kahane et al., 2012). As with emotional empathy, inconsistent evidence has been reported for age-related changes in measures of cognitive empathy like perspective taking or ToM scores, including evidence for stability (Keightley et al., 2006), decline (Bailey et al., 2008; Charlton et al., 2009), and an inverse-*U*-shaped pattern (Phillips et al., 2011; O’Brien et al., 2013) which implies a decrease at least for the late adulthood. A deterioration of cognitive empathy/ToM seems to be partially mediated by the decline of executive functions (Charlton et al., 2009; Phillips et al., 2011) but also partly independent from executive processing (Kemp et al., 2012). In summary, age-related changes of cognitive empathy/ToM are probable and – as outlined above – their influence on alterations of moral decision making reasonable.

On the basis of age-related affective and cognitive changes as well as on preliminary evidence for age-related changes of moral permissibility ratings in late adulthood (Moran et al., 2012), it is likely that alterations of moral decision making also occur with increasing age from young to late adulthood. When evaluating the permissibility of moral transgressions, older adults’ judgments are more strongly driven by outcome than younger adults’ judgments, meaning that older adults less likely consider the intentions of a moral agent (Moran et al., 2012). This result was interpreted in the context of age-related declines in cognitive empathy/ToM and changes in motivational information processing.

Given the knowledge about neuropsychological mechanisms of moral decision making: Why should aging affect this process? In fact, there are at least three arguments: First, age-related changes of affective processing have been reported, which are relevant for moral decision making (e.g., Beadle et al., 2015). Second, changes in cognitive functions that are related to moral decision making have been found to be altered with advancing age (e.g., Borghesani et al., 2013). Third, elderly people show (non-moral) decision making difficulties under conditions of risk and ambiguity that affect their everyday life, because they show problems to estimate risks and are prone to dubious offers (Denburg et al., 2007; Brand and Markowitsch, 2010). Moreover, a moderation of the relationship between age and risky decision making by executive functions and reasoning abilities was reported (Brand and Schiebener, 2013). Based on these arguments, the current study investigates the question, how age-related affective and cognitive changes influence possible age-related alterations of moral decision making.

Recently, first neuroscientific evidence was published that point to sex differences in moral decision making for extreme situations. Fumagalli et al. (2010a) showed that men more often override intuitive moral options in highly emotional and difficult moral dilemmas than women do. This indicates that men decide moral trade-offs rather pragmatically and despite the risk of harm for others whereas women decide more empathically and caring for others at risk (Fumagalli et al., 2010a). Additionally, the frequency of choices that result from overridden intuitive options could be manipulated by transcranial direct current stimulation of the ventromedial prefrontal cortex in women, but not in men (Fumagalli et al., 2010b), indicating that moral decisions appear to be more easily modified by external factors in women whereas men’s moral decisions are more robust against external (emotional) cues (Fumagalli et al., 2010a,b). Youssef et al. (2012) demonstrated that under conditions of stress females tend to make less utilitarian moral decisions than males. However, the outlined data was found for moral decisions made in extreme (not everyday) moral dilemma situations and were not discussed in relation to age. The current study tries to transfer these results to everyday moral decision making and investigates its relation between age, sex and related neuropsychological measures: It has been shown that women score higher on emotional empathy (O’Brien et al., 2013), whereas results for sex differences regarding executive functions (Gur et al., 2012; Kim et al., 2013) and cognitive empathy/ToM (Pavlova, 2009; Ahmed and Miller, 2011) are insconsistent. While no sex differences in executive functions were found by Kim et al. (2013), Gur et al. (2012) report that women outperform men in reasoning speed whereas male show better performance in spatial tests. Sex differences for ToM were found in the Faux Pas test, but not in the Reading the Mind in the Eyes Test (Ahmed and Miller, 2011).

As outlined above, the main affective and cognitive functions that are discussed in the context of moral decision making on the one hand and age-related changes as well as sex differences on the other hand are emotional empathy, (executive) reasoning and cognitive empathy/ToM. Therefore we focus these functions in the current investigation as possible mediators for age and sex effects on moral decision making.

Our hypotheses are: Advanced age and female sex positively influence the proportion of altruistic decisions in everyday moral conflict situations. Given that age and sex have an effect on emotional empathy, and emotional empathy should positively influence altruistic moral decisions, it can be expected that the age and sex effects on moral decision making are mediated by emotional empathy.

Likewise, as both (executive) reasoning and cognitive empathy/ToM have been shown to be related to moral decision making as well as age and sex (although with conflicting results with regard to the direction of these relationships, as described above), we hypothesize that age and sex effects on moral decision making are – in addition to emotional empathy – mediated by executive reasoning and cognitive empathy/ToM.

To test these hypotheses, an everyday moral decision making task that was already used in former studies (Starcke et al., 2011; Rosen et al., 2013) and revised and validated recently (Rosen et al., 2015) as well as an elaborate neuropsychological test battery were applied to healthy participants between 19 and 90 years of age. In order to test the influence of age and sex on moral decision making as well as possible neuropsychological mediators, a structural equation model (SEM) was calculated.

## Materials and Methods

### Procedure

The study was approved by the local ethics committee (Nr. 02-13). All participants gave written informed consent to the study and study procedure. Participants were recruited among employees and students of the University of Vechta as well as their relatives and friends. No financial compensation was given. Because of the complexity of our outcome variable (i.e., moral decision making), at best small to medium effects could be expected in the calculated correlational analyses. On the basis of power analyses, it was determined that at least 175 participants should be tested to achieve a power of 98% for medium effects (*ρ* = 0.30) and a power of 80% for small, but still meaningful effects (*ρ* = 0.21). Within the aimed sample of 175 participants, it was defined that it should preferably consist of balanced subsamples for every decade between 20 (including 18 and 19 year old participants which are adults according to German jurisdiction) and 90 years of age. Besides age between 18 and 90 years and female or male sex, further inclusion criteria were German mother tongue or very good German language skills and intact or sufficiently corrected vision and hearing for the testing procedures. Inclusion criteria were that participants were male or female adults between 18 and 90 years of age with German as their mother tongue or very good knowledge of German language. Additionally, participants were only enrolled in the study, if they gave informed consent to the study. Exclusion criteria were reports of current or past neurologic or psychiatric diseases as well as current alcohol or drug abuse, an age-inappropriate general cognitive state as measured by the DemTect (Kalbe et al., 2004, cut-off: score <13), and severe clinical symptoms of a depression as measured by the German version (Hautzinger et al., 2009) of the Beck Depression Inventory-II (BDI-II, Beck et al., 1996, cut-off: score >28). Participants who showed increased social desirability as indicated by the Social Desirability Scale 17 (SDS-17, Stöber, 2001, cut-off: score >15) were also excluded. This cut-off score was chosen referring to 1.5 standard deviations above the mean score of the fourth validation sample in Stöber (2001), because it best matches the current sample in terms of age and education.

### Participants

A total sample of 197 healthy participants from 19 to 86 years of age (*M* = 45.98, *SD* = 18.39) who met the inclusion criteria were enrolled in the study. The case numbers of the aimed age subsamples included 47 participants (26 female) aged 18–29 years, 32 participants (21 female) aged 30–39 years, 34 participants (21 female) aged 40–49 years, 36 participants (21 female) aged 50–59 years, 20 participants (13 female) aged 60 and 69 years, 21 participants (15 female) aged 70–79 years and seven participants (five female) aged 80 and 89 years. Sociodemographic and neuropsychological characteristics of the study sample are displayed in **Table 1**.

**Table 1:** Sociodemographic characteristics and neuropsychological test results.

			All participants (*N* = 197)		Females (*n* = 122)		Males (*n* = 75)		Sex effects		
Measure	Max.	Range	*M*	*SD*	*M*	*SD*	*M*	*SD*	*t*(195)	*p*	*d*
Sociodemographic variables											
Age (years)		19-86	45.98	18.39	47.02	18.83	44.31	17.65	1.00	0.317	0.15
Education^a^ (years)		7-18	13.41	2.44	13.24	2.27	13.66	2.66	-1.11	0.270	0.17
General cognitive state											
DEMTECT	18	13-18	16.98	1.31	17.12	1.22	16.75	1.43	1.89	0.061	0.28
Depression											
BDI-II	63	0-27	6.96	5.80	7.48	5.96	6.12	5.45	1.60	0.111	0.23
Social desirability											
SDS-17	17	0-15	9.22	3.34	9.46	3.31	8.84	3.37	1.27	0.208	0.19
Intelligence											
MWT-B^b^	–	16-99	72.16	23.16	71.97	23.16	72.48	23.31	-0.15	0.881	0.02
Emotional empathy											
IRI empathic concern	20	7-36	14.10	3.26	14.67	2.46	13.17	4.11	3.20	0.002	0.47
E-Scale Emotional sensitivity	30	6-49	19.24	5.40	20.06	5.51	17.91	4.97	2.77	0.006	0.40
Emotional concern	35	12-35	24.65	4.75	25.80	4.17	22.79	5.07	4.52	<0.001	0.66
Reasoning											
LPS-4 raw score	40	9-38	27.52	4.93	27.04	5.05	28.29	4.67	-1.74	0.084	0.25
LPS-4 normed score^c^	–	10-85	58.64	11.85	58.75	10.40	58.47	13.96	0.17	0.869	0.02
Executive functions											
MCST Correct	48	14-48	40.86	6.44	40.04	6.67	42.19	5.87	-2.37	0.019	0.34
Errors	–	0-22	5.82	4.60	6.42	4.76	4.85	4.20	2.41	0.017	0.34
Perseverations	–	0-12	1.32	2.38	1.54	2.62	0.96	1.90	1.80	0.073	0.24
Key search test	16	0-16	11.49	4.34	11.23	4.59	11.92	3.91	-1.08	0.280	0.16
Cognitive empathy/ToM											
RMET	36	6-34	23.36	5.51	23.45	4.59	23.20	4.40	0.38	0.706	0.06
IRI perspective taking	20	7-36	14.93	3.00	15.07	2.50	14.72	3.67	0.79	0.433	0.12
E-Scale Cognitive sensitivity	25	5-25	15.42	4.77	15.87	4.61	14.69	4.98	1.69	0.093	0.25
Cognitive concern	25	6-25	15.67	3.90	16.22	3.68	14.81	4.10	2.50	0.013	0.37

*Order in the table does not represent the order of task application (which is reported in the procedures section); Max., possible maximum score; BDI-II, Beck Depression Inventory-II; MWT-B, Mehrfach Wortschatz Test-B (German intelligence test, see text); LPS, Leistungsprüfsystem (German intelligence test battery, see text); MCST, Modified Card Sorting Test; IRI, Interpersonal Reactivity Index; ToM, Theory of mind; RMET, Reading the Mind in the Eyes Task; SDS-17, Social Desirability Scale 17 (see text); ^a^sum of school and professional education; ^b^normed percentage scores; ^c^normed T scores*.

### Neuropsychological Test Battery

Besides the screening instruments that were used to exclude cognitive dysfunction and severe depressive symptoms as well as increased social desirability, all participants completed a neuropsychological test battery including instruments to assess intelligence as well as tests to examine functions that are relevant for moral decision making, i.e., emotional empathy, (executive) reasoning and cognitive empathy/ToM.

Intelligence was assessed using the German “Mehrfach Wortschatz Test-B” (MWT-B, Lehrl, 2005) which measures general intelligence.

Emotional empathy was assessed by the “empathic concern” scale of the Interpersonal Reactivity Index (IRI, Paulus, 2009), which assesses the tendency to “experience feelings of compassion” and concern for others (Davis, 1983). Furthermore, the factors “emotional sensitivity” and “emotional concern” of the German version of the E-Scale (Leibetseder et al., 2007) were used, because they represent emotional reactivity to social situations.

Taken from the German intelligence test battery “Leistungsprüfsystem” (LPS, Sturm et al., 1993), the fourth subtest (LPS-4) was applied to examine reasoning abilities. In addition, reasoning was assessed by the measurement of executive functions that are associated to reasoning and cognitive control, i.e., cognitive flexibility and planning (Diamond, 2013). Cognitive flexibility was assessed by a computerized version of the Modified Card Sorting Test (MCST, Nelson, 1976). Planning was measured by the Key Search Test, which was taken from the Behavioral Assessment of the Dysexecutive Syndrome test battery (BADS, Wilson et al., 1996).

Theory of mind was measured using a German version of the “Reading the mind in the Eyes” Test (RMET, Baron-Cohen et al., 2001). Additionally, the “perspective taking” scale from the German version of the IRI (Paulus, 2009), that assesses the ability to adopt other people’s perspective (Davis, 1983) as a measure of cognitive empathy, was used. Furthermore, the E-Scale (Leibetseder et al., 2007) factors “cognitive-sensitivity” and “cognitive-concern” were applied. In this study, established definitions of cognitive empathy/ToM and emotional empathy are used, that regard ToM as the cognitive inference of mental states, and therefore overlaps cognitive facets of empathy, whereas emotional empathy is regarded as the emotional resonance with affective states of others (e.g., Hojat et al., 2011; Kemp et al., 2012). Thus, we include cognitive empathy/ToM as a composite factor into our SEM that represents a domain of overlapping concepts. Based on the data published by the authors of the E-Scale (Leibetseder et al., 2007), the four factors of the E-Scale were divided in two emotional empathy factors and two cognitive empathy factors that were assigned to cognitive empathy/ToM functions as defined above. The “cognitive” empathy factors require active reflection on social situations or change of perspective and therefore fit to the cognitive empathy/ToM definition applied in the current paper, whereas the “emotional” empathy factors represent spontaneous emotional empathizing corresponding to the definition of emotional empathy applied in the current paper.

### Moral Decision Making Task

A revised version of an everyday moral decision making task (Starcke et al., 2011; Rosen et al., 2013) was applied that was validated and already used in its revised form in another study (Rosen et al., 2015). The task consists of 20 short stories which describe moral dilemma situations that could potentially resemble personal experience of the participants or be encountered during the participants’ future life. All situations were designed to cause cognitive conflict and high personal involvement. For each situation, participants have to decide between a morally desirable and a personally preferable behavior. The revised form of the moral decision making task was validated with regard to the nature of the morally desirable (“altruistic”) and personally preferable (“egoistic”) options as well as to their emotionality and their similarity to everyday real-life situations in a previous study (for further details and the complete set of stories please see supplement of Rosen et al., 2015). Items of low, medium and high (statistical) item difficulty were chosen within subsets of high and low emotional stories, so that floor or ceiling effects do not have to be expected.

The total set of short stories is composed of 10 high emotional and 10 low emotional stories that address the reader as the main actor. An example for a high emotional story is: “On the stairs you meet your disliked neighbor who is not feeling well, asking you if you give him a lift to see a doctor. Do you help your neighbor?” An example for a low emotional story is: “In a hurry you arrive at a red pedestrian stoplight, where a school class is already waiting for the light to turn green. Do you cross the street?” The task was presented to the participants as pen and paper questionnaire. All participants were instructed to refer to the first situation that comes to their mind while they read the stories. Hereby, situations would easily be imaginable and would thus provoke high personal involvement and increase ecological validity. At the end of every story, participants were asked a forced choice “yes” or “no” question whether or not they would choose a proposed “egoistic” (personally preferable) or “altruistic” (morally desirable) behavior, if they were part of the described situation. Half of the proposed behaviors were designed to be egoistic (*n* = 10) while the other half (*n* = 10) were altruistic, with five high and five low emotional stories in both sets. Word count and word lengths were matched for all subsets of stories. For high emotional, low emotional, and the whole set of stories, the percentage of altruistic decisions was calculated.

### Statistical Analysis

Standard statistical data analyses were carried out using IBM SPSS Statistics for Windows, Version 20.0 (Armonk, IBM Corp.). Variables were tested for normal distribution using the Kolmogorov–Smirnov (K–S) test. Because of the large sample size (*N* > 30), even small deviations of normal distribution are detected by the K–S test (Cohen et al., 2003; Bortz et al., 2008). In this case, graphical methods were applied to estimate the degree of deviation from normality and to check for possible outliers (e.g., Cohen et al., 2003; Bortz et al., 2008), using histograms, Q–Q plots and boxplots. If deviations from normal distribution were considered to be small and hence biased results were not to be expected, parametric analyses that are robust against normality violation were applied (e.g., Rasch and Guiard, 2004; Bortz et al., 2008).

For interval scaled variables that satisfy the above outlined requirements, independent group differences were tested by the Student’s *t*-test for independent samples, analyses of variance (ANOVA) for more than two groups and analyses of covariance (ANCOVA), if the influence of additional variables was controlled for. Interval scaled within subjects comparisons were conducted by Student’s *t*-test for repeated measures.

For ordinal variables and in case of deviations from normal distribution that were considered to be large enough to bias obtained results or if homogeneity of variance was violated as indicated by Levene’s test, non-parametric analyses were calculated. Independent group differences were calculated with the Mann–Whitney-*U*-Test or Kruskal–Wallis-Test. Differences between nominal variables were examined applying the chi-square (X^2^) goodness of fit test.

Relationships between variables were examined with Pearson product-moment correlations and partial correlations in case of approximately normally distributed variables.

The alpha level was set to 5% for all tests and Bonferroni correction for multiple testing was applied where necessary. All tests were performed two-tailed. Cohen’s *d* effect sizes for univariate between-group differences were calculated using pooled standard deviation.

The SEM analysis was carried out using MPlus 6 (Muthén and Muthén, 2011). There was no missing data. Latent variables were indicated by two manifest variables that were chosen based on theoretical considerations outlined above. For mediator analyses, indicator variables were required to correlate with age and sex as predictors and moral decision making as outcome variable (Baron and Kenny, 1986). The model fit was evaluated using standard criteria (Hu and Bentler, 1995, 1999): The standard root mean square residual (SRMR; values below 0.08 indicate a good fit with the data), comparative fit indices, i.e., the Comparative Fit Index (CFI) and Tucker Lewis Index (TLI; values above 0.90 acceptable fit; values above 0.95 indicate excellent fit), and the root mean square error of approximation (RMSEA; “test of close fit”; a value below 0.08 with a significance value below 0.05 indicates acceptable fit). Confidence intervals (CI) are reported where appropriate.

## Results

Sociodemographic data and neuropsychological test results are shown in **Table 1**. All cognitive measures were in the normal range. General intelligence was measured slightly above average, while reasoning performance as measured by the LPS-4 was average. A total of 27.1% of all participants exhibited mild or very mild depressive symptoms.

### Moral Decision Making and Age

Age was significantly associated with overall moral decision making (*r* = 0.24, *p* < 0.001) indicating that altruistic decisions increase with age (**Table 2**). Moreover, age was significantly related to low emotional (*r* = 0.29 *p <* 0.001), but not high emotional (*r* = 0.08, *p* = 0.247) moral decisions (**Table 2**) indicating that the aging effect in overall moral decisions can primarily be traced back to decisions in low emotional dilemma situations.

**Table 2:** Correlations between age, moral decision making scores and neuropsychological variables and intercorrelations between moral decision making scores.

Variable	Age (in years)		Overall moral decision making^a^		High emotional moral decision making^a^		Low emotional moral decision making^a^	
	*r*	*P*	*r*	*p*	*r*	*p*	*r*	*p*
Moral decision making^a^								
All dilemma stories	0.24	<0.001	–	–	0.76	<0.001	0.86	<0.001
High emotional stories	0.08	0.247	0.76	<0.001	–	–	0.35	<0.001
Low emotional stories	0.29	<0.001	0.86	<0.001	0.35	<0.001	–	–
Emotional empathy								
IRI empathic concern	0.10	0.163	0.36	<0.001	0.33	<0.001	0.26	<0.001
E-Scale Emotional sensitivity	-0.19	0.007	-0.03	0.715	-0.02	0.772	-0.02	0.770
Emotional concern	0.23	0.001	0.36	<0.001	0.26	<0.001	0.32	<0.001
Reasoning								
LPS-4 raw score	-0.43	<0.001	-0.15	0.035	-0.07	0.312	-0.16	0.022
LPS-4 normed score^b^	-0.01	0.872	0.13	0.070	0.15	0.033	0.07	0.329
Executive functions								
MCST Correct	-0.45	<0.001	-0.18	0.011	-0.07	0.345	-0.21	0.003
Errors	0.43	<0.001	0.16	0.027	0.05	0.518	0.19	0.007
Perseverations	0.37	<0.001	0.18	0.010	0.09	0.192	0.20	0.006
Key search test	-0.35	<0.001	-0.11	0.133	0.03	0.638	-0.18	0.010
Cognitive empathy/ToM								
RMET	-0.29	<0.001	-0.02	0.788	0.02	0.735	-0.04	0.543
IRI perspective taking	-0.01	0.941	0.18	0.013	0.14	0.057	0.15	0.037
E-Scale Cognitive sensitivity	-0.19	0.007	0.00	0.993	-0.02	0.772	-0.02	0.770
Cognitive concern	0.12	0.101	0.17	0.019	0.14	0.058	0.14	0.049

*Reported *p*-values are uncorrected; LPS, Leistungsprüfsystem (German intelligence test battery, see text); MCST, Modified Card Sorting Test; IRI, Interpersonal Reactivity Index; ToM, Theory of mind; RMET, Reading the Mind in the Eyes Task; ^a^Percentage of altruistic decisions; ^b^normed T scores*.

Participants made significantly more altruistic decisions in high than in low emotional moral situations, *t*(196) = 5.34, *p* < 0.001. Results of the moral decision making task and sex effects are listed in **Table 3**.

**Table 3:** Moral decision making in the overall sample as well as in female and male participants.

Variable			All participants (*N* = 197)		Females (*n* = 122)		Males (*n* = 75)		Sex effects		
	Max.	Range	*M*	*SD*	*M*	*SD*	*M*	*SD*	*t*(195)	*p*	*d*
Moral decision making^a^											
All dilemma stories	100	25–95	67.37	14.15	68.89	13.77	64.91	14.50	1.93	0.055	0.28
High emotional stories	100	20–100	71.20	14.85	71.56	14.38	70.53	15.67	0.47	0.640	0.07
Low emotional stories	100	20–100	63.42	19.91	66.11	19.47	59.04	19.65	2.47	0.014	0.36

*^a^Percentage of altruistic decisions*.

### Relationships between Age, Moral Decision Making and Variables of Empathy, Executive Functions and Cognitive Empathy/ToM

Correlations between age and moral decision making scores with measures for emotional empathy, executive functions, and cognitive empathy/ToM were calculated as prerequisite for the intended mediation analyses (**Table 2**). Age was significantly correlated with reasoning operationalized with the LPS-4 four raw score, emotional empathy as measured by the E-Scale scores emotional sensitivity and emotional concern, cognitive flexibility and planning as operationalized with the MCST and the Key search test, and cognitive empathy/ToM as assessed with the RMET and the cognitive sensitivity factor of the E-Scale. Intercorrelations between moral decision making scores are also listed in **Table 2**.

Depending on the applied moral decision making score (overall, high emotional, low emotional), altruistic moral decisions were differentially related to reasoning, emotional empathy, and executive functions (**Table 2**): Overall altruistic moral decisions were related to emotional empathy and executive functions (i.e., cognitive flexibility as measured by correct answers and perseverative errors in the MCST), whereas low emotional altruistic moral decisions were related to reasoning (LPS-4), emotional empathy, and executive functions (i.e., cognitive flexibility and planning as measured by correct answers and perseverative and non-perseverative errors in the MCST and the Key Search Test). Correlations between low emotional moral decision making and cognitive empathy/ToM as measured by the IRI perspective taking score and the E-Scale cognitive concern score failed significance after correction for multiple comparisons (**Table 2**). Altruistic moral decision making in high emotional situations was only related to emotional empathy. Intercorrelations between neuropsychological variables are listed in **Table 4**. These show that emotional empathy measures are intercorrelated, related to the cognitive empathy/ToM scores, and are correlated with the LPS raw score which is not controlled for age effects. The LPS raw score is also related to cognitive empathy/ToM scores as well as to executive functions. The age-controlled LPS norm score, however, is only correlated with measures of executive functions which supports our integration of these measures to a composite operationalization of “executive reasoning.” Finally, ToM as measured by the RMET is not correlated to cognitive empathy questionnaire measures, except for the E-Scale cognitive sensitivity factor. Therefore a conceptual overlap is indicated. No other significant correlations were found.

**Table 4:** Intercorrelations between neuropsychological variables.

Variable	IRI empathic concern	E-Scale emotional sensitivity	E-Scale emotional concern	LPS-4 raw score	LPS-4 normed score^a^	MCST correct	MCST errors	MCST perseverative errors	Key search test	RMET	IRI perspective taking	E-Scale cognitive sensitivity	E-Scale cognitive concern
Emotional empathy													
IRI empathic concern	-	0.11	0.48***	-0.15*	0.05	-0.07	0.06	0.08	-0.10	-0.04	0.28***	0.14	0.36***
E-Scale Emotional sensitivity	0.11	-	0.08	0.02	0.00	0.01	-0.07	0.11	0.11	0.10	0.15*	0.65***	0.24**
Emotional concern	0.48***	0.08	-	-0.24**	-0.05	-0.14	0.11	0.17*	-0.11	-0.04	0.30***	0.19**	0.65***
Reasoning													
LPS-4 raw score	-0.15*	0.02	-0.24**	-	0.45***	0.48***	-0.46***	-0.41***	0.16*	0.23**	0.15*	-0.01	-0.17*
LPS-4 normed score^a^	0.05	0.00	-0.05	0.45***	-	0.20**	-0.19**	-0.16*	0.04	0.02	-0.05	-0.05	-0.03
Executive functions													
MCST Correct	-0.07	0.01	-0.14	0.48***	0.20**	-	0.96***	-0.85***	0.29***	0.28***	0.12	-0.02	-0.19**
Errors	0.06	-0.07	0.11	-0.46***	-0.19**	-0.96***	-	0.67***	-0.30***	-0.27***	-0.12	0.00	0.17*
Perseverations	0.08	0.11	0.17	-0.41***	-0.16*	-0.85***	0.67***	-	-0.22**	-0.24**	-0.08	0.04	0.17*
Key search test	-0.10	-0.11	-0.11	0.16*	0.04	0.29***	-0.30***	-0.22**	-	0.23**	-0.06	0.11	0.01
Cognitive empathy/ToM													
RMET	-0.04	0.10	-0.04	0.23**	-0.02	0.28***	-0.27***	-0.24**	0.23**	-	0.10	0.18*	0.00
IRI perspective taking	0.28***	0.15*	0.30***	0.15*	-0.05	0.15	-0.12	-0.08	-0.06	0.10	-	0.18*	0.14*
E-Scale Cognitive sensitivity	0.14	0.65***	0.19**	-0.01	-0.05	-0.02	0.00	-0.04	0.11	0.18*	0.18*	-	0.38***
Cognitive concern	0.36***	0.24**	0.65***	-0.17*	-0.03	-0.19*	0.17*	0.17*	0.01	0.00	0.14*	0.38***	-

*Reported *p*-values are uncorrected; LPS, Leistungsprüfsystem (German intelligence test battery, see text); MCST, Modified Card Sorting Test; IRI, Interpersonal Reactivity Index; ToM, Theory of mind; RMET, Reading the Mind in the Eyes Task; ^a^normed T scores. ^∗^*p* < 0.05. ^∗∗^*p* < 0.01. ^∗∗∗^*p* < 0.001*.

### Sex Effects on Neuropsychological Measures and Moral Decision Making

Female and male participants did not differ in terms of age and education or any neuropsychological test scores except for higher cognitive flexibility in men with medium effect sizes and higher emotional empathy scores in women with large effect sizes (**Table 1**).

Female and male participants’ moral decisions significantly differed in low emotional moral decisions with a medium effect size indicating that women made more altruistic moral decisions (**Table 3**).

For overall moral decision making and high emotional moral decisions, no significant differences between women and men were found.

### Mediation of Age and Sex Effects on Moral Decision Making

A SEM was calculated with age and sex (dummy-coded with 1 = female and 2 = male sex) as predictors, emotional empathy and reasoning as hypothesized mediators and moral decision making as dependent variable. For latent variables, two manifest variables were chosen that fit the construct on theoretical considerations outlined above and highly correlated with both, age and moral decision making. Because none of the applied cognitive empathy/ToM measures was correlated with both age and moral decision making, cognitive empathy/ToM could not be included in the SEM.

Therefore, only emotional empathy and reasoning were introduced to the SEM and measured on a latent level. Emotional empathy was represented as latent variable by the manifest variables IRI empathic concern and E-Scale emotional concern score, because they fit to the applied construct of emotional empathy and exhibited the highest correlations with moral decision making and were substantially correlated with age. The latent variable reasoning was measured by the manifest variables correct trials of the MCST and the raw score of the LPS-4. Correct trials of the MCST were chosen, because cognitive flexibility is hereby represented by successful set-shifting operations, and the raw score of the LPS-4 was chosen, because it measures logical reasoning abilities that are not controlled for age differences. Both variables were additionally highly correlated with age and moral decision making.

We included low emotional moral decisions as dependent variable in the SEM for two reasons: Firstly, only this subscale was correlated significantly with age, emotional empathy and executive reasoning. Likewise, sex differences were only significant for low emotional but not high emotional moral decisions. Therefore, the requirements for mediation analyses were fulfilled only for the low emotional moral dilemmas. Secondly, the overall score of the moral decisions was not used, although there were some significant correlations with other variables of interest, because the low emotional moral decisions contribute 50% to the total score. Given that the two subscales of the moral dilemmas were differently correlated with other variables, it would be inappropriate to sum up the scores and use this as one dependent variable in the SEM.

**FIGURE 1:**
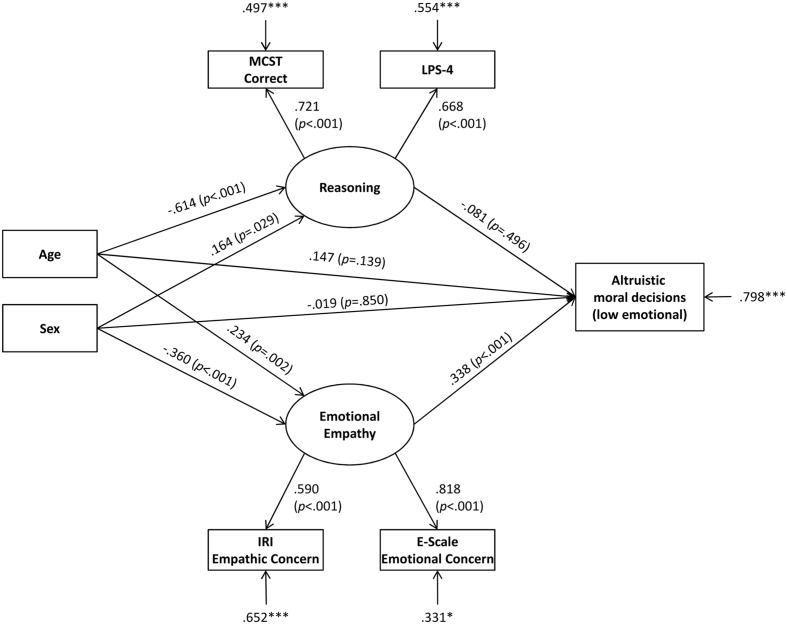
**Results of the structural equation model including factor loadings of the latent dimensions, β -weights, *p*-values, and residuals**. ^∗^*p* < 0.05, ^∗∗∗^*p* < 0.001.

The results show that the positive relationship between age and altruistic moral decisions in low emotional situations as well as sex differences are fully mediated by emotional empathy, but not by reasoning. The proposed theoretical model fitted the data very well: The RMSEA was lower than 0.001 with a 90% CI of [0.000, 0.082] and a PCLOSE (probability that the RMSEA is lower than 0.05) of 75.3%. The CFI was 1.000, the TLI was 1.003, and the SRMR was 0.026, all indicating an excellent fit. The *X*^2^ test was not significant, *X*^2^= 7.76, *p* = 0.457, which means that the theoretical model fits to the empirical data. In summary the theoretical model fitted the data excellently. A significant proportion of the variance in moral decision making was explained by the SEM (*R*^2^= 0.202, *p* = 0.001). The mediation model with all direct and indirect effects is shown in **Figure 1**. In this model, the direct effects of age and sex on moral decision making were not significant. The direct effects of age and sex on reasoning and emotional empathy were significant. The positive β-weight for the effect of sex on reasoning means male sex is associated to better reasoning, while the negative β-weight for the effect of sex on emotional empathy means female sex is associated to better emotional empathy. For the latent variables, only the direct effect of emotional empathy on moral decision making was significant with higher emotional empathy promoting more altruistic moral decisions.

The indirect effects of age and sex on moral decision making were not significant over reasoning (age: β = 0.050, *p* = 0.498; sex: β = -0.013, *p* = 0.517), but over emotional empathy (age: β = 0.079, *p* = 0.022; sex: β = -0.122, *p* = 0.009). This means that only emotional empathy, but not reasoning, mediates significantly age and sex effects on moral decision making.

### Additional Analyses

Given the bivariate correlations between the subtests representing reasoning and moral decision making (low emotional dilemmas), but no direct effect of reasoning on moral decision making in the SEM, we tested a reduced model to inspect these results further. Firstly, we calculated the SEM without the mediator emotional empathy. In this model, we again found a significant effect of age on reasoning (on latent level) with β = -0.615, *p* < 0.001, but no direct effect of reasoning on moral decision making (β = -0.115, *p* = 0.348). Interestingly, the direct effect of age on moral decision making remained significant (β = 0.205, *p* = 0.041) in this reduced model without emotional empathy as a mediator. Sex had also a significant effect on reasoning (β = 0.164, *p* = 0.029), but the direct effect of sex on moral decision making slightly failed to reach significance (β = -0.135, *p* = 0.053). Secondly, we also calculated partial correlations between the two subtests of reasoning and moral decision making (low emotional dilemmas) controlled for age. These partial correlations were not significant (MCST correct *r* = -0.096, *p* = 0.182; LPS-4 *r* = -0.048, *p* = 0.504).

## Discussion

The current study examined effects of age and sex on moral decision making and their mediation by emotional empathy, (executive) reasoning and cognitive empathy/ToM functions. We found main effects of (higher) age and (female) sex on altruistic moral decision making on a bivariate level, but these direct effects were fully mediated by emotional empathy in the SEM. Reasoning, although also correlated with both the predictors and the dependent variable on a bivariate level, did not mediate the effect of age and sex on moral decision making significantly. Lastly, even though theoretically hypothesized, cognitive empathy/ToM could not be included in the SEM, because no cognitive empathy/ToM subscale correlated significantly with both moral decision making and age on a bivariate level. In summary, our hypotheses of positive relationships between altruistic moral decision making and age as well as sex were confirmed, and our mediation hypotheses were confirmed for emotional empathy.

Therefore, the current data indicate that individual performance of age- and sex-related moral decision making is established by age and sex effects on emotional empathy, i.e., that altruistic moral decision making is related to greater levels of emotional empathy in older people and people of female sex. This finding is interesting and brings up two central questions: Why do advanced age and female sex predict higher emotional empathy scores? Why is emotional empathy mediating the relationship between age/sex and altruistic moral decisions? In addition, it is also worth noting that reasoning did not mediate the effect of age and sex on moral decision, although it was correlated with all these variables on a bivariate level, and that cognitive empathy/ToM was not systematically correlated with age and moral decisions. These issues are discussed in detail below.

### Emotional Empathy

In line with former studies (e.g., Hein et al., 2011; Van der Graaff et al., 2014), our results underpin the central role of emotional empathy in altruistic moral decision making. As a possible explanation, Batson (2010) proposed that empathic concern motivates altruistic behavior and hereby highlights prosocial goals. Interestingly, calculating the mediation of the observed age and sex effects on altruistic moral decisions was only possible for the low emotional moral decisions as dependent variable, but not for the high emotional moral decisions. This was caused by the differential correlation pattern for low vs. high emotional moral decisions. While all requirements for mediation analyses were fulfilled for the low emotional moral decisions, the high emotional decisions were unrelated to age and sex and to most of the other variables included in the SEM. Thus, obviously the low and high emotional moral decisions tap into different psychological processes. It might be argued that, as high emotional moral situations evoke higher emotional arousal (Starcke et al., 2011), these decisions are driven by strong emotional empathic reactions and moral intuitions (Haidt, 2001; Batson, 2010; Hein et al., 2011) which might be not susceptible to relatively subtle age- and sex-related differences in emotional empathy (in contrast to decisions in low emotional dilemmas). These altruistic, less “flexible” intuitions are considered to derive from an ontogenetically early developed “moral sense” that is founded on the development of neural pathways such as the hypothalamic-limbic axis and their connections to the prefrontal cortex as well as the internalization of social norms and values, both influenced by parents, peers and communities (Narvaez and Lapsley, 2009). Consequently, strong aversive affective reactions are needed to interfere with high emotional moral decisions, as it has been shown for aversive affective states that are induced through stress (Starcke et al., 2011). Furthermore, it was shown that egoistic moral decisions are accompanied by aversive affective states (Sommer et al., 2010) – making it less likely to overcome these in high emotional dilemmas. This effect is reflected in our data, given that the mean scores of altruistic decisions in the high emotional dilemmas were descriptively higher with a smaller variance than the scores in the low emotional dilemmas, and sex effects were not shown at all for the high emotional stories. Thus, in low emotional dilemmas, a less intuitive decision is more likely, so that here, age- and sex-related levels of empathic concern facilitates altruistic moral decisions (Beadle et al., 2015).

Although the existing literature on age-related increases in emotional empathy is controversial (May and Alligood, 2000; Bailey et al., 2008; Grühn et al., 2008; O’Brien et al., 2013), the observed positive age effect on emotional empathy in our study is consistent with the results of Gould and Macneil Gautreau (2014), who found higher empathic concern in older as compared to younger adults. The increase of emotional empathy with advancing age and its importance for moral decisions can be interpreted in the light of the socioemotional selectivity theory (Carstensen et al., 1999; Carstensen, 2006) which posits that with increasing age – and with the increasing awareness of the finiteness of the own life – there is a motivational shift toward more affective and especially more positive stimuli (also called the “positivity effect”), whereas negative affective information is actively avoided (e.g., Charles et al., 2009). This change in motivation is also regarded to be associated with the relatively stable emotional well-being with increasing age in spite of cognitive and physical losses (e.g., Mather et al., 2012). It also leads to preferences for social interactions that are emotionally gainful, rather than serving other goals such as information seeking. In this sense, higher emotional empathy may be one central function that enables elderly people to approach these preferred social interactions or behaviors and avoid negative affective reactions that arise from moral violations (Sommer et al., 2010). This interpretation is in line with the finding that induced emotional empathy facilitates prosocial behavior in older adults stronger than in younger adults (Beadle et al., 2015). Altruistic behavior, i.e., actions away from the own egoistic interests and toward social concern and communal welfare, can be interpreted as one expression of this behavior, induced by emotional empathy. This should be particularly the case in dilemmas which are not emotionally described and of high impact for the own person.

Our finding of higher emotional empathy in women than in men is consistent with broader evidence for such sex effects in all ages from adolescence through late adulthood (O’Brien et al., 2013; Van der Graaff et al., 2014). It also fits with results that women exhibit a higher likelihood for emotional contagion and a greater intensity of emotional experiences in social situations assessed by self-report and electrophysiological measures (Brody and Hall, 2008). Furthermore, women typically show greater kindness and consideration for others’ needs than men (e.g., compassion for cheated persons) and are more likely to help and share and less likely to harm others (Jaffee and Hyde, 2000; Brody and Hall, 2008).

The relationship of sex and moral decision making, mediated by emotional empathy, could reflect recent findings about greater effort that women put in the prevention of harm in moral conflicts, in which women more than men searched for additional options beyond the scope of the conflict’s immediate context (Migliore et al., 2014). Thus, more emotional empathy in women – and probably other processes related to the consideration of other persons’ interests and worrying about a positive outcome for them – accounts for the fact that more altruistic decisions are made and that woman typically show a stronger “moral sense” (Fumagalli et al., 2010a) than men.

Sex effects regarding emotional empathy and in consequence, also moral decisions, are – at least partly – consistent with sex-related social roles, goals and motives (Brody and Hall, 2008; Fumagalli et al., 2010a), given that many of the moral dilemmas used in our study include social interactions. Culturally shaped stereotypes and socialized sex roles contribute to the outlined effects because girls’ socialization is more often related to responsibility for others’ well-being (e.g., as child caretaker) and boys’ socialization more often is related to self-assertion, power and status (e.g., as economic provider; Brody, 1997; Fumagalli et al., 2010a). Furthermore, personality factors as well as cognitive, affective and situational factors contribute to sex differences in socio-emotional processing (Brody, 1997) which may have an impact on moral decision making (e.g., Haviv and Leman, 2002). Also beyond sex roles, biological factors may account for the sex effects in moral decision making (Fumagalli et al., 2010a,b; Youssef et al., 2012). Integrating these arguments, the sex effects on decision making in everyday moral dilemmas may be explained by biological factors (like dispositions and sex-related neural differences), together with socialization and culturally shaped sex roles, which are additionally considered important for self-reported emotional empathy. This notion is corroborated by developmental studies focusing on childhood development of social cognition and morality (e.g., Decety et al., 2011; Van der Graaff et al., 2014).

Taken together, it appears plausible that emotional empathy mediated the observed age- and sex-related increase of altruistic moral decisions. Our data give evidence for increasing emotional empathy with advancing age (evidenced for an age range from 18 to 86 years and thus for a time span from early to late adulthood) and higher emotional empathy in women and the central function of this higher empathic concern for altruistic moral decision making.

### Reasoning

Consistent with many previous studies (e.g., Head et al., 2009; Hodzik and Lemaire, 2011; Borghesani et al., 2013), we found a very strong effect of age on reasoning, operationalized by the subtest 4 of the LPS and the MCST. However, contradictory to our hypotheses, we did not find a mediating effect of reasoning on the relationship between age and moral decision making, and even the direct effect from reasoning to moral decision making was not significant in the SEM (even not in the reduced SEM without emotional empathy as mediator). This was the case, although there were significant correlations between both subtests of reasoning and moral decision making on a bivariate level. The additional analyses indeed showed that these bivariate correlations did not remain significant when being controlled for age in partial correlations, indicating that age, but not reasoning, is the relevant variable that can explain variance in moral decision making. And these age effects are mediated by emotional empathy, but not by reasoning.

The effect of reasoning on moral decision making was expected, because altruistic intuitions that promote situationally proposed behaviors might be overridden for the pursuit of personal aims by means of reasoning and executive control (Greene et al., 2004; Kahane et al., 2012; Paxton et al., 2012). Given that reasoning has been proposed to be related to the resolution of cognitive conflict, which is an ingredient of solving moral dilemmas (Greene et al., 2004; Patterson et al., 2012), cognitive control functions should theoretically have an impact on moral decision making *per se* and on the relationship between age and moral decision making in particular. Our current data do not represent these theoretical arguments. We have to notice, however, that the structure of the reasoning tasks used in the current study only covers certain subprocesses of executive functioning and cognitive conflict monitoring. It seems worth investigating in future studies whether reasoning and executive components, which are more closely related to conflict processing and decision making in general, may explain additional variance of moral decision making across the lifespan.

In the same direction, we found effects of sex on reasoning, but the indirect effect of sex on moral decision making was not significant, which is caused by the non-significant direct effect of reasoning on moral decision making (see above). It should be noted that our findings regarding sex effects on reasoning and executive functions is inhomogenous, as performance in planning, as operationalized by the key search test, and raw scores in the LPS 4 did not differ between men and women. However, this pattern reflects previous studies showing inconsistent results with either no effects (e.g., Kim et al., 2013) or small effects in very specific cognitive-executive domains with advantages for women typically in verbal cognitive and pair association tasks and for men in processing speed and visuospatial processing (e.g., Portin et al., 1995). Hereby, it is suggested that sex effects in executive performance depend on the executive function that is measured. Obviously, sex-specific reasoning functions do not add substantial information to understanding the effects of sex on moral decision making.

In summary, emotional empathy mediates the age and sex effects on moral decision making, whereas executive reasoning does not mediate these effects, although it was related to egoistic moral decisions on a bivariate level. However, it seems too early to give a final statement on the assumption that age and sex effects on moral decision making can be explained independent of reasoning abilities, because reasoning should theoretically influence the process of moral decision making in many ways, such as emotion regulation, cognitive reflection, and *post hoc* justification (Haidt, 2001; Patterson et al., 2012; Paxton et al., 2012). The present study investigated executive reasoning abilities represented by logical reasoning, cognitive flexibility, and planning performance without testing their specific application to the moral problem itself. Furthermore, other executive funtions that were not tested in the curent study might influence moral confict processing, such as problem solving, interference control and response inhibition (Patterson et al., 2012; Diamond, 2013). The role of reasoning and other executive functions might therefore be underestimated and further studies are needed which assess specifically how reasoning skills are applied to moral problems and additionally use a broader range of tests assessing specific cognitive functions and reasoning abilities.

### Cognitive Empathy/Theory of Mind

Cognitive empathy/ToM was associated to age, but not to moral decision making. The RMET and the cognitive sensitivity factor of the E-Scale, which were aggregated to a composite cognitive empathy/ToM domain, were negatively related to age, but not to moral decision making. Therefore, cognitive empathy/ToM could not be included in our mediation model, because the requirements of bivariate correlations among the predictor, the mediators and the dependent variable were not fulfilled. As indicated by the intercorrelations of ToM and cognitive empathy measures, a possible reason for our finding that different cognitive empathy/ToM measures relate to age as opposed to moral decision making can be deduced by the fact that ToM and cognitive empathy were measured by the different tasks. The RMET is a power test and assesses the capability of ToM usage. However, the RMET does not inform us about the extent to which ToM is applied in different contexts. In contrast, the self-report questionnaire measures of cognitive empathy (i.e., the IRI perspective taking score and the cognitive sensitivity factor of the E-Scale) express the readiness to use ToM functions and the extent to which ToM is applied to appropriate situations. This issue cannot be resolved by the current data and needs further clarification in future studies. In the present SEM, potential mediation effects of cognitive empathy/ToM in the context of age, sex and moral decision making could not be resolved due to insufficient prerequisites and therefore remain issues for further studies with a more elaborative test battery assessing cognitive empathy/ToM skills.

Considering the correlational results, the observed age-related decline in cognitive empathy/ToM is in line with previous reports of diminished cognitive empathy/ToM in old age, which is discussed to be partly related to cognitive-executive decline (e.g., Charlton et al., 2009; Phillips et al., 2011). Further it fits to age-related social motivational changes as described above as well as evidence that moral permissibility judgments in older adults are more likely outcome driven than in younger adults (Moran et al., 2012).

Contradicting the observed correlations between cognitive empathy/ToM and altruistic moral decision making that slightly failed significance, cognitive empathy/ToM was found associated to more frequent egoistic decisions in a previous study investigating everyday moral decisions in a sample of adults with a mean age of 62 years (Rosen et al., 2013). However, these assumingly inconsistent relations are established by different cognitive empathy/ToM measures, the IRI perspective taking score and the E-Scale cognitive concern score in the present study and the RMET in the former study. Therefore, cognitive empathy/ToM might be used differently in the context of moral decision making (e.g., depending on the applied cognitive empathy/ToM measure) and it may depend on how meaningful social contextual information are accounted for (Baez et al., 2012), e.g., other people’s intentions (Young et al., 2010; Killen et al., 2011).

On the basis of our results, one might have the impression that cognitive empathy/ToM is unsystematically related to both age and moral decision making. Given that cognitive empathy/ToM is a multi-dimensional construct (Adolphs, 2001), these results are preliminary and a more detailed definition of cognitive empathy/ToM subfunctions supposed to be related to both age-sensitive changes and moral decision making should be the basis for further research.

### Limitations

There are several limitations that influence the results of the current investigation. First there are only few tests that represent emotional empathy, executive reasoning, and cognitive empathy/ToM. Executive reasoning was measured by tests that assess logical reasoning, cognitive flexibility and planning, whereas other components like problem solving, response inhibition are not measured. Emotional empathy and cognitive empathy/ToM are mostly assessed by questionnaire measures. Future studies should use an elaborative test battery assessing these functions in more detail and with more tasks in order to have a full SEM on latent level including all theoretically plausible mediators. In addition, further tasks assessing moral dilemmas are needed in order to also include the dependent variable on latent level in the SEM.

The fact that only one moral decision making paradigm was used in the current study did not only limit the modeling of the dependent variable on manifest level, but also reduce the generalization of the findings. Future studies should compare everyday moral decisions to extreme moral dilemma situations and how emotional empathy, executive reasoning, and cognitive empathy/ToM potentially contribute to making decisions in both paradigms. Moral permissibility ratings on moral transgressions could also be included to investigate the influence of the abovementioned functions on a third person observer’s appraisal.

Interesting variables that probably influence moral decision making and need further investigation are moral intuitions (Graham et al., 2011), that have been shown to be altered in criminal offenders (Aharoni et al., 2011), and affective states that might influence moral decisions like anxiety, anger, or guilt (Polman and Ruttan, 2011), because lower levels of anger and anxiety in older adults have recently been reported (Shallcross et al., 2013).

Due to the cross-sectional design of the study, intra-individual changes of moral decision making cannot be described, and the age-related changes observed might be the result of cohort effects. Age-related effects on emotional empathy, for example, have earlier been interpreted as cohort effects rather than genuinely developmental effects (Grühn et al., 2008; O’Brien et al., 2013). However, the reported cohort effects in O’Brien et al. (2013) are inconsistent with the current findings, as younger cohorts in that study showed higher emotional empathy, whereas older cohorts showed lower emotional empathy scores. In contrast, the older cohorts in our study scored higher on emotional empathy measures. Future research with longitudinal study designs, which allow to identify intra-individual changes, may clarify these issues.

Finally, it has to be considered that the obtained sex differences are found for uneven sample sizes. We have investigated a rather large total sample; however, the ratio of women and men was approximately 1.5:1. This might have biased the obtained sex effects. Consequently, sex effects on neuropsychological functions remain a topic of discussion. Furthermore, the impact of different socialization and sex roles can be a biasing cohort effect. Future research should address these issues.

## Conclusion

The current study shows that age and sex effects on moral decision making are mediated by emotional empathy. In other words, older age and female sex increase the probability of being more empathic and this explains more altruistic moral decision making. Thus, age and sex differences converge to higher emotional empathy abilities that determine the frequency of altruistic moral decisions. The level of emotional empathy which older adults and/or adults of female sex more likely show than younger and/or male adults is more crucial for the promotion of altruistic moral decisions than age or sex themselves.

Further research is needed to investigate in which way emotional empathy and possible other neuropsychological functions interact with moral decision making in the context of everyday and extreme moral conflicts as well as moral permissibility ratings. Also, the role of moral attitudes and affective states like anxiety, anger and guilt in the context of age- and sex-effects on moral decision making needs future investigations.

Moral decision making is an essential component of communal life and human societies on the whole. Commitment to social prescriptions as well as flexible behavioral adjustment to social challenges are necessary elements of a person’s ability to manage social intercourse on the one hand, and advocate personal interests on the other hand. Our results may help to explain uneven age and sex distributions concerning specific characteristics of western societies, for example in volunteer work and social and economic professions as well as in forensic statistics (Fumagalli et al., 2010a). Furthermore, they may contribute to debates on social inclusion and socio-emotional self-actualization in an aging society.

## Author Contributions

JBR contributed to conception and design of the work; acquisition, analysis, and interpretation of data; drafting and revising the work and gave final approval of the version to be published. JBR agrees to be accountable for all aspects of the work. MB contributed to the analysis and interpretation of data for the work; drafting and revising the work and gave final approval of the version to be published. MB agrees to be accountable for all aspects of the work. EK contributed to conception and design of the work; acquisition, analysis, and interpretation of data; drafting and revising the work and gave final approval of the version to be published. EK agrees to be accountable for all aspects of the work.

## Conflict of Interest Statement

The authors declare that the research was conducted in the absence of any commercial or financial relationships that could be construed as a potential conflict of interest.
